# Editorial: Long-term care for older people: A global perspective

**DOI:** 10.3389/fpubh.2023.1178397

**Published:** 2023-04-11

**Authors:** Bo Hu, Ricardo Rodrigues, Raphael Wittenberg, YongJoo Rhee

**Affiliations:** ^1^Care Policy and Evaluation Centre, London School of Economics and Political Science, London, United Kingdom; ^2^ISEG Lisbon School of Economics and Management, SOCIUS—Research Centre in Economic and Organizational Sociology/CSG—Research in Social Sciences and Management, Lisboa, Portugal; ^3^Department of Health Sciences, Dongduk Women's University, Seoul, Republic of Korea

**Keywords:** older people, healthy aging, care provider, quality of life, long-term care, informal care

## Introduction

Long-term care is essential to the quality of life of older people. It enables older people to maintain a level of functional capability consistent with their basic rights, freedoms, and human dignity. As population aging continues to accelerate across the world, meeting the rising demand for long-term care is set to be a global challenge facing many societies. Not only should the long-term care system of a country be sustainable and efficient in care production in the long run, but it must also ensure the adequacy and quality of care, promote distributive fairness for every member of society, and contribute to broader societal aims such as gender equality.

A thorough investigation of long-term care for older people is a fundamental step if we want to build a sustainable and resilient long-term care system. This Research Topic aims to promote a better understanding of the demographic, social, and economic dimensions of long-term care utilization and provision and their implications for policies and the wellbeing of older people and their families. So far, much of the debate, policy interventions and empirical evidence on long-term care have centered on high-income countries, with the exception of transnational care where care workers in low and middle-income countries migrate to high-income countries to provide care. However, by 2050, an estimated 80% of older people will be living in middle and low-income countries (1). There are nine studies in this Research Topic with contributing authors from diverse geographical contexts and academic disciplines. A wide range of topics is covered by those studies.

## Key issues in long-term care for older people

In the global context of population aging, a shortage of labor force in the long-term care sector is an urgent issue many countries need to address. Two studies in the Research Topic have looked into this issue. Roland et al. focused on reasons for turnover and absenteeism among personal assistants (PAs) in England. They found that distance traveled to work and number of PAs employed by individual employers are strongly associated with sick leave taken by PAs. Based on evidence from England, Teo et al. studied the relationship between turnover, hiring and employment growth in the long-term care sector. Their analyses demonstrate that care worker turnover and recruitment are negatively related, and that rising vacancies are associated with a decline in employment.

Effective and high-quality service delivery is the key to creating a positive and supportive environment for those using long-term care. Rosteius et al. studied Green Care Farms as innovative and alternative long-term care environments in comparison to regular nursing homes in the Netherlands. They argued that how a care organization is designed significantly impacts residents' daily life and their mental, physical and social functioning. They called for leaders in the long-term sector to rethink existing ways of care delivery. The COVID-19 pandemic posed great challenges to service delivery for older people living in care homes. As older people were disproportionately affected by the pandemic, governments faced considerable difficulties in identifying and implementing effective protective measures for older care home residents. Das observed that, in the case of Hong Kong, China, this was further complicated by poorly designed indoor environments in care homes with inadequate ventilation and cramped spaces. The author argued that fundamental reforms to healthcare systems, updating the antiquated regulations, and continued optimization of the built environment are needed to turn long-term care facilities into positive spaces for caregiving.

Family caregivers will continue to play an indispensable role across different countries in the foreseeable future. In high-income countries such as the US, although formal care increased over the decades, informal caregivers are still crucial in terms of supporting the long-term care sector (2). In some low-income countries, long-term care responsibilities are almost exclusively assumed by family caregivers. Caregivers may face especially challenging tasks when they provide care for older people with certain health conditions such as dementia, stroke, or cancer. As such, a better understanding of the challenges and consequences associated with family caregiving is vitally important. In this Research Topic, research conducted by Nia et al. focused on developing and validating the Care Challenge Scale (CCS) for family caregivers of people with Alzheimer's disease in Iran. The results of the confirmatory factor analyses show that their 10-item scale has good validity, internal consistency, and stability in the Iranian long-term care setting that can be used by therapists, nurses, and researchers to assess the challenges faced by this group.

It is notable that more countries have in recent years treated increasing the capacity of formal care services as a government priority. A case in point is mainland China. Zhang et al. reported that mainland China has witnessed a rapid increase in government policies relating to formal long-term care services in the past decade. On examining the care policies developed by the Chinese government between 2011 and 2019, they found that the comprehensiveness and consistency of policy instruments are associated with rising capacity in care institutions. This shows different paths of policy development when compared with, for example, Europe, where the stated aim has been enhancing aging in place.

China has introduced pilot long-term care insurance schemes in 29 cities across the country since 2016. Drawing on data collected in the city of Guangzhou, Peng et al. found that people's understanding of and satisfaction with long-term care insurance policies are associated with heightened trust in those policies. Liu et al. developed a set of service capability indicators for long-term care facilities in China. Based on a Delphi consultation, their index system comprised 31 individual indicators across six subdimensions including staffing, facilities and equipment, funding, medical inspection services, health management services, and institutional standards.

Building capacity for formal care services is a direct response to the expected increase in demand for long-term care in the coming decades, to ensure that care needs in the older population can be adequately met. The international literature has shown increased attention to and interest in unmet long-term care needs. Based on latent profile analyses, Cao et al. demonstrate that unmet long-term care needs are significant risk factors for poor health in the Chinese older population. Their study points to the important role of adequate levels of care in promoting healthy aging.

## Conclusion

Long-term care policy is a multi-faceted issue that calls for multi-stakeholder solutions. Rising demand for care and labor shortages prompt policymakers, caregivers, service users, and researchers to rethink the existing models of care delivery and to map out pathways for preventing unmet needs and care poverty in the older population. Governments should make sure that sufficient funding and financial investment are put in place to support caregivers and people with care needs. This is a fundamental issue if a country wants its long-term care system to function satisfactorily. However, addressing the challenges associated with long-term care is not only about funding. Identifying and monitoring the dynamics of care needs in the older population, timely delivery of high-quality services, fully utilizing the power of modern caregiving technology, and promotion of healthy aging all contribute to a well-developed and sustainable long-term care system. Policies that protect the health and wellbeing of formal and informal caregivers also play an essential role. At the heart of this issue is the need for a governance model that facilitates communication and encourages collaboration between different stakeholders.

## Author contributions

BH drafted the first version of the editorial. RR, RW, and YR drafted, edited, and revised the editorial. All authors contributed to and approved the submission of the editorial.

## References

[1] World Health Organisation. Ageing and Health. Geneva: World Health Organisation (2022).

[2] Van Houtven CH Konetzka RT Taggert E Coe NB. Informal and formal home care for older adults with disabilities increased, 2004–16: study examines changes in the rates of informal home care use among older adults with disabilities 2004 to 2016. Health Aff. (2020) 39:1297–301. 10.1377/hlthaff.2019.01800 32744936 PMC8729158

